# Regime-aware causal Bayesian forecasting for non-stationary time series

**DOI:** 10.1371/journal.pone.0356474

**Published:** 2026-08-21

**Authors:** Brandon Mossop, Salimur Choudhury

**Affiliations:** School of Computing, Queen’s University, Kingston, Ontario, Canada; Feng Chia University, TAIWAN

## Abstract

Non-stationary time series are common in many real-world domains, including infectious disease spread, where the underlying relationships between variables evolve over time. However, most existing forecasting methods assume stationarity and fail to capture changing causal dynamics. To address this challenge, we propose the Causal Regime Bayesian (CaReBayes) forecasting framework, which integrates regime detection, causal discovery, and Bayesian forecasting within a unified approach. CaReBayes segments time series into regimes using temporal causal discovery, fits a Bayesian structural autoregressive model for each regime, classifies the current regime, then performs regime-specific forecasting with uncertainty quantification. The framework introduces methodological advances: a grid-search procedure for automated regime-dependent causal discovery, a classification method that assigns future observations to regimes based on learned Bayesian structures, and regime-conditioned Bayesian structural forecasting. Across both synthetic and Ontario COVID-19 time series data, CaReBayes outperforms benchmark models for time series forecasting. In addition to improved forecasting performance, it produces regime-dependent causal graphs that summarize candidate structural relationships in the system, enhancing interpretability.

## Introduction

When we observe time series (measurements indexed by time at regular intervals), we often ask: What will happen in the future (forecasting)? Another equally important, and often more difficult, question is *why* these time series behave as they do (interpretability). Both questions become significantly more challenging when the time series are *non-stationary*, meaning that the statistical properties of the system evolve over time.

Common forms of non-stationarity in time series are *trends*, when a time series increases or decreases over time, and *seasonality*, when repeating patterns occur within a fixed time interval. Another important form of non-stationarity arises when the *causal structure* of a time series evolves over time. Within the structural causal model (SCM) framework, the causal structure of data is represented as a directed acyclic graph (DAG) [1]. In time series, the DAG captures both temporal self-dependencies (self-lags) and causal relationships between different variables. If the data-generating process can be described by a single, stable causal graph, the system is said to be *causally stationary*. However, when the causal structure itself evolves, the system exhibits *causal non-stationarity*.

Causal non-stationarity is pervasive in domains such as finance, epidemiology, and engineering [2], where changes in policy or seasonal effects often induce distinct *regimes*, intervals during which the joint distribution of variables remains internally consistent but changes across time. A time series is therefore *regime-dependent* when it remains stationary within contiguous intervals (regimes) but exhibits structural changes between them, which may also recur.

Standard forecasting methods often assume stationarity or only account for changes in distributions, potentially missing structural shifts in causal relationships. Existing work on regime-dependent forecasting includes Markov-switching Vector AutoRegressive (MS-VAR) models [3,4], which allow dynamics to vary across latent regimes, and structural time series models [5], which model evolving components such as trends and seasonality. Threshold-based autoregressive models also address regime dynamics by segmenting time series based on observed threshold variables; for instance, a subset threshold autoregressive framework was developed to select lag terms, delay parameters, and threshold configurations [6]. Similarly, hysteretic autoregressive models allow transitions to depend on both a transition variable and the preceding regime, requiring stronger evidence to trigger a regime shift and preventing rapid switching near boundary limits [7]. In the machine learning (ML) literature, forecasting approaches address non-stationarity through adaptation and concept drift detection [8,9], while switching dynamical systems [10] capture latent state transitions.

To uncover evolving relationships, causal discovery methods have been adapted to non-stationary data by estimating causal graphs across regimes or over time [11,12]. This line of work has also been extended to include forecasting. For example, a state-space framework has been proposed in which causal relationships evolve continuously over time with latent processes, enabling joint causal discovery and forecasting in nonstationary environments [13].

In real-world applications, such as tracking infectious disease transmission, a parallel body of work focuses on spatial and spatiotemporal modeling. Prior work includes a Bayesian framework for spatial integer-valued time series [14], as well as comparative analysis of spatial-temporal hurdle and zero-inflated GARCH models for dengue fever case counts [15]. These models effectively address geographic dependencies, spatial heterogeneity, and excess zero counts.

Despite these advances, an important gap remains between methods that model regime-dependent statistical dynamics and methods that identify changing causal structure. Markov-switching models allow dynamics to vary across latent regimes, but do not estimate regime-specific causal graphs. Threshold and hysteretic models provide transition mechanisms, but their regimes are defined through observed transition variables and threshold boundaries rather than changes in causal structure. Switching dynamical systems and state-space models can represent evolving latent processes, but many non-stationary systems instead exhibit abrupt, discrete, and potentially recurring regime changes [3]. Moreover, state-space methods can be difficult to extend to high-dimensional settings [16]. Non-stationary causal discovery methods can identify changes in causal structure, but do not provide a complete framework for classifying future observations into causal regimes and producing regime-conditioned forecasts with posterior predictive uncertainty. Spatiotemporal infectious-disease models address geographic and count-valued dependence, but do not address regime-dependent temporal causal discovery.

To bridge this gap, we introduce CaReBayes (pronounced “Care Bayes”), a unified framework for Causal Regime Bayesian Forecasting that jointly performs regime detection, causal structure learning, regime classification, and Bayesian autoregressive forecasting. Furthermore, unlike threshold and hysteretic approaches, CaReBayes does not require the prior specification of an observed threshold variable or fixed threshold boundaries. Instead, it identifies regimes through changes in estimated temporal causal structure and classifies new observations according to their predictive compatibility with learned regime-specific structural models.

To our knowledge, CaReBayes is the first framework to combine discrete regime-dependent temporal causal discovery, predictive-error-based classification of future observations into learned causal regimes, and regime-conditioned Bayesian structural forecasting. The framework aims to support causal understanding by identifying candidate drivers of a target variable while also providing point forecasts and posterior predictive uncertainty.

## Materials and methods

To evaluate the forecasting performance of our proposed CaReBayes framework, we compare it against two widely used linear forecasting baselines. The first, the Vector Autoregressive (VAR) model, assumes a stationary process and serves as a regime-agnostic benchmark. The second, the Markov-Switching VAR (MS-VAR) model, allows for regime-dependent dynamics governed by a latent Markov process but remains purely statistical (non-causal).

### Benchmark models

Because the synthetic datasets used in this study are generated from linear processes, we restrict all baselines to linear models for fairness and interpretability [17].

#### Vector autoregressive model (VAR).

The Vector Autoregressive model (VAR) represents each time series variable as a linear combination of its own past values and those of all other variables. It assumes that the data-generating process is stable over time. Model estimation was performed with Maximum Likelihood Estimation under Gaussian noise assumptions.

The VAR model does not account for structural changes or regime shifts. It provides a baseline for evaluating methods that are regime-dependent such as our proposed CaReBayes framework and the Markov-Switching VAR model.

#### Markov-switching VAR (MS-VAR).

The Markov-Switching Vector Autoregressive (MS-VAR) model [3] extends the VAR framework by allowing parameters to vary across a finite number of latent regimes. These regimes are governed by a first-order Markov process, meaning the active regime at any time depends probabilistically on the previous one.

Each regime has its own intercepts, autoregressive coefficients, and noise structure, enabling the model to capture sudden or recurring changes in system dynamics. Parameters and regime probabilities are estimated iteratively using the Expectation–Maximization (EM) algorithm combined with the Hamilton filter for forward-backward inference.

While MS-VAR can identify the regime patterns statistically, it does not capture causal relationships among variables or provide interpretable uncertainty quantification. In contrast, CaReBayes explicitly discovers regime-specific causal structures and performs Bayesian forecasting conditioned on those causal regimes.

### Regime-aware causal Bayesian framework (CaReBayes)

**Fig 1:**
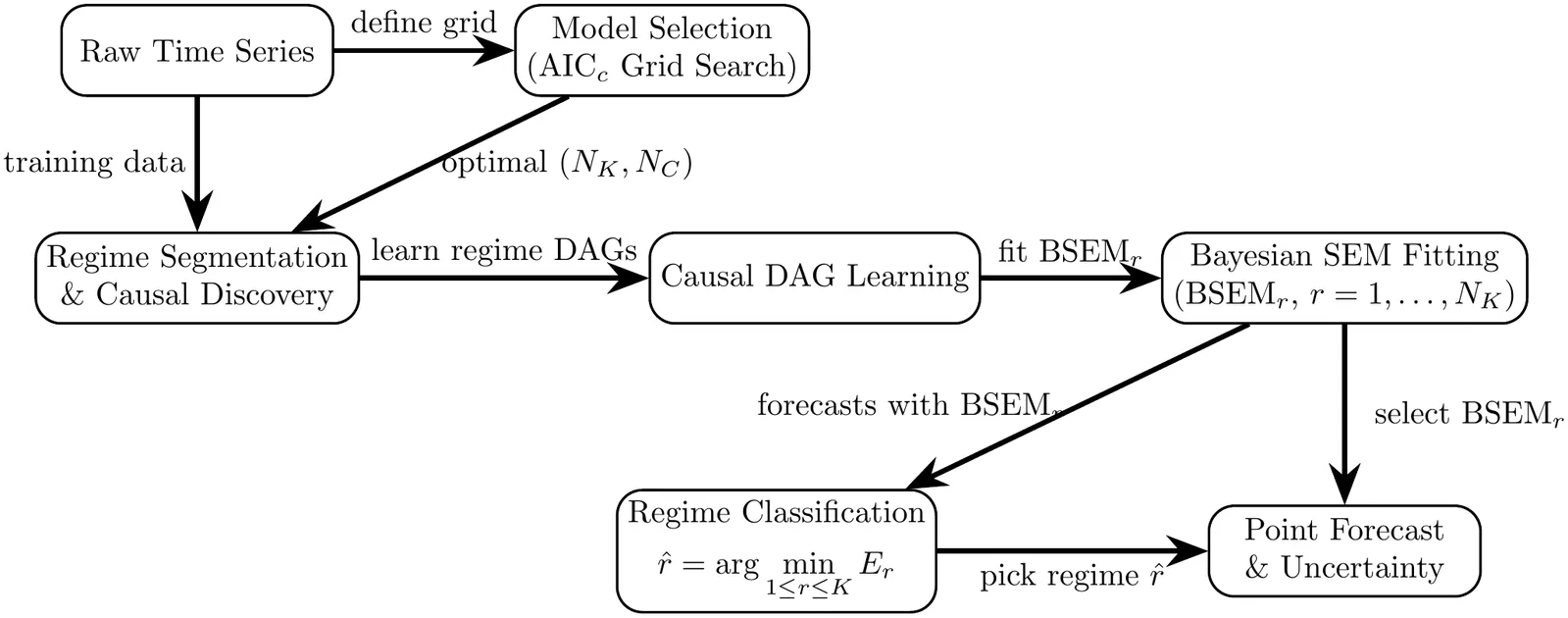
Overview of the CaReBayes framework. The CaReBayes framework integrates regime detection, causal discovery, and Bayesian forecasting. Each regime is identified through causal graph segmentation, and a Bayesian structural autoregressive model is fit to each regime to enable regime-aware forecasting with uncertainty quantification.

This section describes the proposed CaReBayes framework and the specific methods used for each component. As illustrated in Fig 1, the approach can be described in four stages: (1) regime segmentation with causal discovery, (2) regime-specific Bayesian structural autoregressive modeling, (3) regime classification, and (4) forecasting with uncertainty quantification.

#### Regime segmentation and causal discovery.

To estimate regime-dependent causal structures, we use the Regime Peter-Clark Momentary Conditional Independence (RPCMCI) algorithm [12], an extension of PCMCI [16] adapted for non-stationary time series.

PCMCI is a constraint-based causal discovery algorithm for time series. Consider a system with *d* time series ${𝐗}_{t}=(X_{t}^{1},...,X_{t}^{d})$ evolving as


$$\begin{array}{c} X_{t}^{j}=f_{j}(Pa(X_{t}^{j}),\eta_{t}^{j}), \end{array}$$

where $Pa(X_{t}^{j})$ denotes the causal parents and $\eta_{t}^{j}$ the noise variable of $X_{t}^{j}$. The parents $Pa(X_{t}^{j})$ form a sufficient conditioning set that induces conditional independence, in accordance with the causal Markov property [18].

PCMCI consists of two stages: (1) the *PC*_1_ algorithm, which performs an iterative condition-selection procedure to identify a set of candidate parents for each variable by testing conditional independencies with increasing conditioning set sizes, and (2) MCI, which refines these candidates by testing momentary conditional independence. This tests whether a candidate parent $X_{t-\ell }^{i}$ (where ℓ is the lag) is conditionally independent of $X_{t}^{j}$ given the selected parents of $X_{t}^{j}$ and $X_{t-\ell }^{i}$, removing indirect and spurious links.

The causal interpretation used here is therefore structural rather than predictive. Granger causality determines whether past values of a variable improve prediction of another variable given the available information set. By contrast, PCMCI attempts to identify a lagged parent set by testing conditional independencies implied by a structural causal model. Thus, a link $X_{t-\ell }^{i}\to X_{t}^{j}$ is interpreted as a candidate structural causal parent under the assumptions of the causal Markov condition, faithfulness, causal sufficiency, temporal ordering, and correct specification of the lag window and conditional independence test. In practice, these assumptions are strongest in the simulated experiments, where the data-generating process is known, and weaker in the observational COVID-19 application.

RPCMCI extends this framework to non-stationary settings by detecting changes in conditional independencies over time and estimating a causal graph for each regime. In this study, we use a linear partial correlation test as the conditional independence criterion, producing a causal window graph for each regime segment.

Regime discovery depends on the number of regimes ($N_{K}$) and transitions ($N_{C}$), which are selected automatically through a grid search guided by the corrected Akaike Information Criterion (AICc):


$$\begin{array}{c} AICc=-2\log L+2N_{\text{para}}+\frac{2N_{\text{para}}(N_{\text{para}}+1)}{T_{\text{eff}}-N_{\text{para}}-1}, \end{array}$$ (1)

where logL is the model log-likelihood, *N*_para_ is the number of estimated parameters, and $T_{\text{eff}}=T-\ell$ is the effective sample size with maximum lag ℓ (set to 1 in all experiments).

The AICc criterion is used only for selecting the discrete regime configuration, namely the number of regimes $N_{K}$ and the number of regime transitions $N_{C}$. This step is separate from the Bayesian estimation of the regime-specific forecasting models, which is discussed next. After the optimal $\left(N_{K},N_{C}\right)$ configuration is selected, CaReBayes conditions on this regime structure and estimates the parameters of each regime-specific structural autoregressive model using Bayesian linear regression. Thus, the framework combines likelihood-based model selection for the discrete regime structure with Bayesian posterior estimation and posterior predictive forecasting conditional on the selected structure.

A limitation of this approach is that uncertainty in the selected regime configuration is not propagated into the posterior predictive distribution. The reported forecast uncertainty therefore reflects parameter and observation uncertainty within the selected regime structure, but not uncertainty over alternative $\left(N_{K},N_{C}\right)$ configurations. Bayesian alternatives could place priors over the number of regimes and transition locations and compare candidate regime structures using marginal likelihood or Bayes factors.

If we let $r\in \{1,...,N_{K}\}$ index regimes, then for each regime *r*, we define a regime-specific causal graph $G_{r}$. The parent set of node $X_{t}^{j}$ in regime *r* is denoted by


$$\begin{array}{c} {Pa}_{r}(X_{t}^{j})=\{X_{t-\ell }^{i}\}, \end{array}$$

where *i* indexes variables and ℓ indexes time lags. The total number of parameters is given by


$$\begin{array}{c} N_{\text{para}}=(N_{K}-1)N_{C}+\sum_{r=1}^{N_{K}}\sum_{j=1}^{d}\left|{Pa}_{r}(X_{t}^{j})\right|. \end{array}$$ (2)

The optimal pair $\left(N_{K},N_{C}\right)$ is selected as the configuration minimizing AICc across all candidates.

#### Bayesian structural autoregressive modeling.

After regime segmentation, CaReBayes fits a Bayesian structural autoregressive model to each regime, encoding the causal relationships discovered by RPCMCI. An autoregressive model expresses each variable at time *t* as a function of its lagged values.

For each regime *r*, every variable $X_{t}^{j}$ is modeled as a function of its causal parents plus noise:


$$\begin{array}{c} X_{t}^{j}=f_{r}({Pa}_{r}(X_{t}^{j}))+\eta_{t,r}^{j}, \end{array}$$ (3)

where $f_{r}(\cdot )$ is an unknown function and $\eta_{t,r}^{j}$ denotes regime-specific noise. In this study, we assume a linear relationship, a single lag (ℓ=1) and Gaussian noise, so that


$$\begin{array}{c} X_{t}^{j}=\sum_{i\in {Pa}_{r}(X_{t}^{j})}\beta_{ir}^{j}X_{t-1}^{i}+\eta_{t,r}^{j},\;\eta_{t,r}^{j}\sim 𝒩(0,\sigma_{j,r}^{2}). \end{array}$$ (4)

with each variable expressed as a linear combination of its causal parents, and $\beta_{ir}^{j}$ quantifies the strength of the causal relationship from variable *i* to *j* within regime *r*. The noise term captures unexplained variability. Model parameters are estimated using Bayesian linear regression, producing posterior distributions over $\beta_{ir}^{j}$ and $\sigma_{j,r}^{2}$ within each regime.

#### Regime classification.

For new observations, CaReBayes infers the active regime ${\hat{r}}_{t}$ by comparing predictive errors across regime-specific models. For each regime *r*, a one-step-ahead prediction ${\hat{𝐗}}_{t}^{\left(r\right)}$ is computed using information up to time t−1, and compared to the observed value ${𝐗}_{t}$. The squared error is then


$$\begin{array}{c} e_{r}(t)=\|{𝐗}_{t}-{\hat{𝐗}}_{t}^{\left(r\right)}{\|}^{2}. \end{array}$$ (5)

Under a Gaussian residual assumption, minimizing $e_{r}(t)$ is equivalent to maximizing the likelihood of ${𝐗}_{t}$ under regime *r*. To improve robustness, errors are aggregated over a rolling window of size *w*, ${𝒲}_{t}=\{t-w+1,...,t\}$:


$$\begin{array}{c} E_{r}(t)=\sum_{s\in {𝒲}_{t}}e_{r}(s). \end{array}$$ (6)

The active regime is then assigned as


$$\begin{array}{c} {\hat{r}}_{t}=\text{arg}\min_{r}E_{r}(t). \end{array}$$ (7)

Regime classification is performed sequentially using observations until time *t*. The inferred regime ${\hat{r}}_{t}$ is then used to generate a *k*-step-ahead forecast for ${𝐗}_{t+k}$. Consequently, the data used for regime inference (i.e., ${𝐗}_{t}$) is distinct from the data used for forecast evaluation (i.e., ${𝐗}_{t+k}$), ensuring that no future information is used during prediction and avoiding any double use of data.

In the experiments, the rolling window size *w* was selected to produce continuous regime segments, ensuring that detected regimes remained stable and continuous over time.

#### Forecast generation and uncertainty quantification.

Once the active regime ${\hat{r}}_{t}$ has been estimated, forecasts are generated using the corresponding regime-specific Bayesian structural autoregressive model. Given the observed data up to time *t*, ${𝐗}_{1:t}$, predictions for future time s*t*eps are obtained from the posterior predictive distribution:


$$\begin{array}{c} p({𝐗}_{t+k}|{𝐗}_{1:t},\Theta_{{\hat{r}}_{t}}), \end{array}$$ (8)

where $\Theta_{{\hat{r}}_{t}}$ denotes the posterior parameters associated with the inferred regime.

In our experiments, forecasts are generated by sampling from this posterior predictive distribution. The mean of these samples provides a point forecast, while the quantiles of the samples define the posterior predictive intervals, allowing uncertainty in future predictions to be quantified.

### Experiments

To evaluate the effectiveness of the CaReBayes framework, we conducted experiments on both synthetic and real-world time series datasets. The synthetic experiments provide access to ground-truth causal structures and regime transitions, allowing us to quantitatively assess its ability to recover true regime-dependent causal graphs and detect regime changes. To further validate its practical applicability, we also applied CaReBayes to a real-world multivariate COVID-19 dataset from Ontario, Canada.

For both the simulated and real-world datasets, all benchmark and proposed models were trained using a lag of one to ensure consistency in the learning setup. In the simulation study, this choice also reflects the true underlying dependency structure, which has a lag of one.

We begin with the simulation studies, which are essential for examining the conditions under which CaReBayes performs well and for evaluating its accuracy in estimating causal regime-dependent dynamics.

#### Simulated data.

The simulation studies evaluate three aspects of the proposed framework: (1) recovery of the true number of regimes ($N_{K}$), regime transitions ($N_{C}$), (2) causal edges, and (3) regime classification accuracy on test data.

Data are generated from a linear structural causal model with regime-dependent parent sets ${Pa}_{r}(X_{t}^{j})$ and first-order lags (ℓ=1). Each system consists of two regimes and has *X*^2^ as the forecasting target.

**Fig 2:**
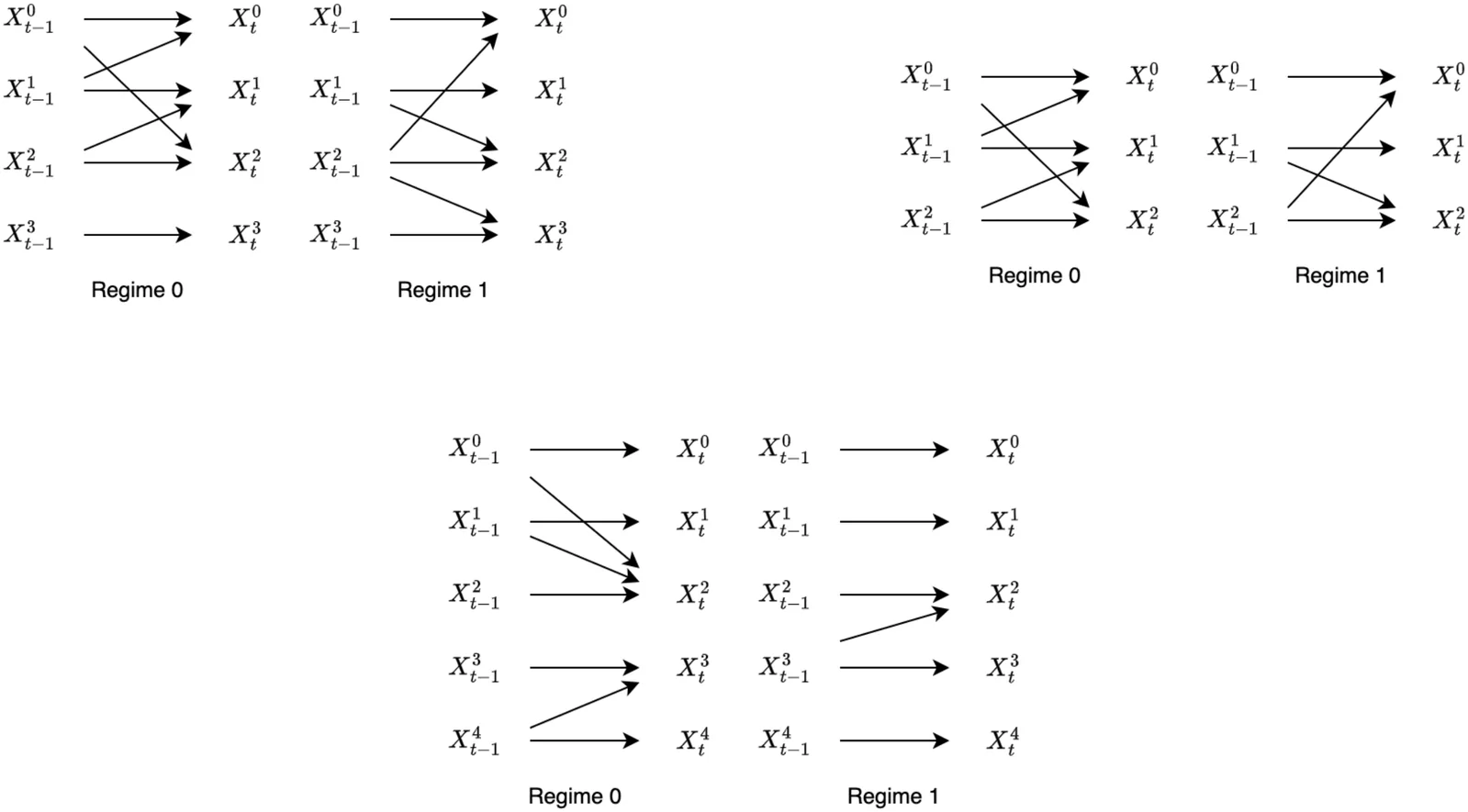
Regime-dependent window causal structures across three simulated systems. (top left) System 2, (top right) System 3, and (bottom) System 1.

A *window* causal graph $G_{r}$ represents the causal structure within each regime. The systems are causally non-stationary, with distinct regime-specific graphs governing the data over time. The causal structures for each system are shown in Fig 2.

Each variable $X_{t}^{j}$ of the simulated data is generated by the following structural equation


$$\begin{array}{c} X_{t}^{j}=\sum_{i\in {Pa}_{r}(X_{t}^{j})}\beta_{ir}^{j}X_{t-1}^{i}+\eta_{t,r}^{j},\;\eta_{t,r}^{j}\sim 𝒩(0,1), \end{array}$$ (9)

where r∈{0,1} denotes the active regime, ${Pa}_{r}(X_{t}^{j})$ is the regime-specific parent set of $X_{t}^{j}$, and the noise terms $\eta_{t,r}^{j}$ are independent across variables, time steps, and Monte Carlo realizations.

System 1 contains five variables and is designed to test whether the proposed framework can recover a substantial change in the causal parents of the target variable $X_{t}^{2}$ across regimes. In Regime 0, $X_{t}^{2}$ depends on its own lag and on the lagged values of $X_{t}^{0}$ and $X_{t}^{1}$. In Regime 1, this mechanism changes: the effect of $X_{t}^{0}$ and $X_{t}^{1}$ on $X_{t}^{2}$ is removed, and $X_{t}^{3}$ becomes the main lagged driver of $X_{t}^{2}$.


$$\begin{array}{c} \begin{array}{cc} \text{Regime }0 & \text{Regime }1\\ \begin{array}{cc} X_{t}^{0} & =0.4X_{t-1}^{0}+\eta_{t,0}^{0},\\ X_{t}^{1} & =0.9X_{t-1}^{1}+\eta_{t,0}^{1},\\ X_{t}^{2} & =0.9X_{t-1}^{2}+0.1X_{t-1}^{0}\\ & \;+0.2X_{t-1}^{1}+\eta_{t,0}^{2},\\ X_{t}^{3} & =0.3X_{t-1}^{3}+0.7X_{t-1}^{4}+\eta_{t,0}^{3},\\ X_{t}^{4} & =0.4X_{t-1}^{4}+\eta_{t,0}^{4} \end{array} & \begin{array}{cc} X_{t}^{0} & =-0.4X_{t-1}^{0}+\eta_{t,1}^{0},\\ X_{t}^{1} & =-0.3X_{t-1}^{1}+\eta_{t,1}^{1},\\ X_{t}^{2} & =0.3X_{t-1}^{2}-1.0X_{t-1}^{3}+\eta_{t,1}^{2},\\ X_{t}^{3} & =-0.3X_{t-1}^{3}+\eta_{t,1}^{3},\\ X_{t}^{4} & =-0.5X_{t-1}^{4}+\eta_{t,1}^{4} \end{array} \end{array} \end{array}$$ (10)

System 2 contains four variables and represents a more moderate regime-dependent change in causal structure. The target $X_{t}^{2}$ remains autoregressive in both regimes, but its additional parent changes across regimes. In Regime 0, $X_{t}^{2}$ depends on $X_{t-1}^{0}$, whereas in Regime 1 it depends on $X_{t-1}^{1}$. This system evaluates whether the method can distinguish regimes when the target mechanism changes, but the overall autoregressive structure remains similar.


$$\begin{array}{c} \begin{array}{cc} \text{Regime }0 & \text{Regime }1\\ \begin{array}{cc} X_{t}^{0} & =0.4X_{t-1}^{0}+0.3X_{t-1}^{1}+\eta_{t,0}^{0},\\ X_{t}^{1} & =0.4X_{t-1}^{1}+0.3X_{t-1}^{2}+\eta_{t,0}^{1},\\ X_{t}^{2} & =0.4X_{t-1}^{2}+0.3X_{t-1}^{0}+\eta_{t,0}^{2},\\ X_{t}^{3} & =0.5X_{t-1}^{3}+\eta_{t,0}^{3} \end{array} & \begin{array}{cc} X_{t}^{0} & =0.4X_{t-1}^{0}+0.3X_{t-1}^{2}+\eta_{t,1}^{0},\\ X_{t}^{1} & =0.4X_{t-1}^{1}+\eta_{t,1}^{1},\\ X_{t}^{2} & =0.4X_{t-1}^{2}+0.3X_{t-1}^{1}+\eta_{t,1}^{2},\\ X_{t}^{3} & =0.4X_{t-1}^{3}+0.3X_{t-1}^{2}+\eta_{t,1}^{3} \end{array} \end{array} \end{array}$$ (11)

Finally, system 3 contains three variables with a single dominant regime change affecting the forecasting target. In Regime 0, $X_{t}^{2}$ is positively driven by $X_{t-1}^{0}$, while in Regime 1, this relationship is replaced by a negative effect from $X_{t-1}^{1}$. This system tests whether the framework can recover regime-dependent changes of the target variable in a lower-dimensional setting.


$$\begin{array}{c} \begin{array}{cc} \text{Regime }0 & \text{Regime }1\\ \begin{array}{cc} X_{t}^{0} & =0.4X_{t-1}^{0}+0.3X_{t-1}^{1}+\eta_{t,0}^{0},\\ X_{t}^{1} & =0.3X_{t-1}^{1}+0.6X_{t-1}^{2}+\eta_{t,0}^{1},\\ X_{t}^{2} & =0.3X_{t-1}^{2}+0.7X_{t-1}^{0}+\eta_{t,0}^{2} \end{array} & \begin{array}{cc} X_{t}^{0} & =0.4X_{t-1}^{0}-0.5X_{t-1}^{2}+\eta_{t,1}^{0},\\ X_{t}^{1} & =0.3X_{t-1}^{1}+\eta_{t,1}^{1},\\ X_{t}^{2} & =0.3X_{t-1}^{2}-0.8X_{t-1}^{1}+\eta_{t,1}^{2} \end{array} \end{array} \end{array}$$ (12)

The systems differ in the number of regime transitions: System 1 has $N_{C}=3$, System 2 has $N_{C}=2$, and System 3 has $N_{C}=1$, with all systems having $N_{K}=2$. Thus, the simulation design evaluates the proposed framework across systems that vary in dimensionality, causal structure, target mechanism, and regime-transition complexity. To assess robustness, each system is simulated across fifty independent Monte Carlo realizations. Performance metrics are reported as means with standard deviations across simulations.

#### Real-world COVID-19 data.

**Fig 3:**
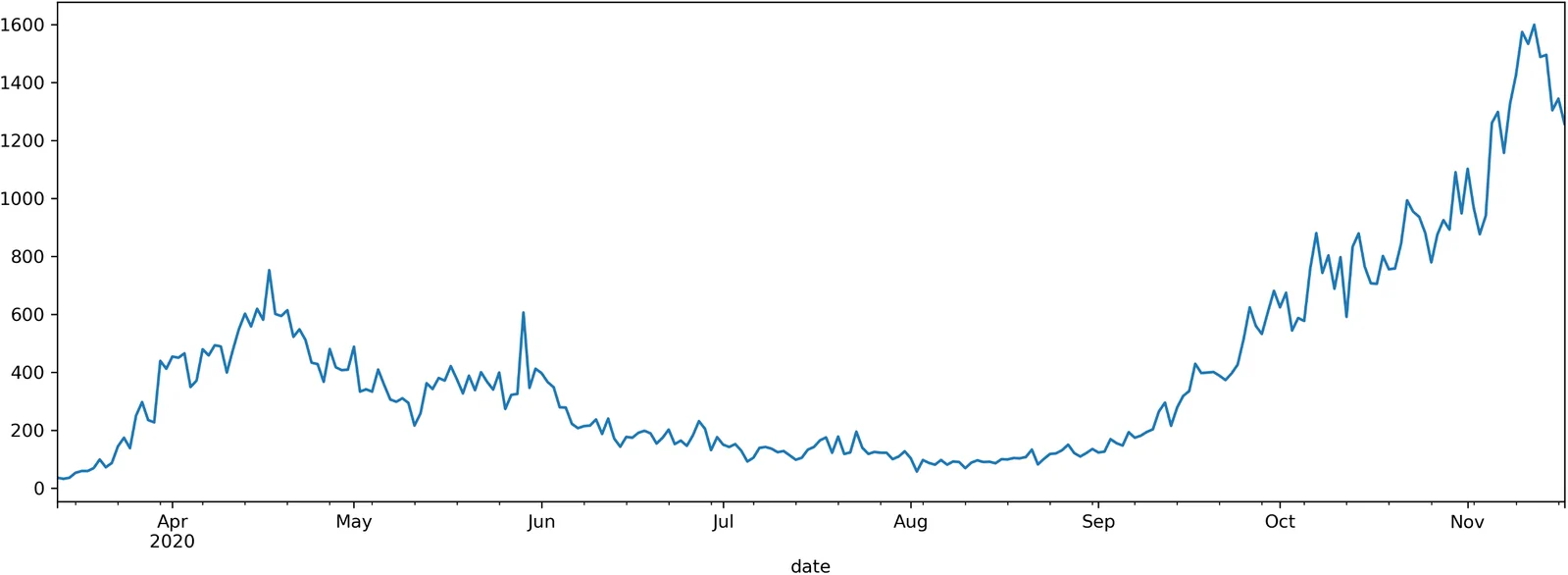
Daily number of new COVID-19 cases in Ontario, Canada from March 13, 2020 to November 17, 2020.

Next, we consider real-world data to evaluate CaReBayes in a practical setting. We focus on time series collected in Ontario, Canada from March 13, 2020 to November 17, 2020 (see Fig 3). Epidemiological data, such as the spread of COVID-19, often exhibits structural shifts that represent distinct regimes [19,20]. The causal structure driving the evolution of the time series can change across regimes.

The CaReBayes framework is well-suited for such applications since it estimates regime-dependent causal structures, classifies future data into a regime, and generates forecasts with both point predictions and uncertainty quantification. This enables interpretable and adaptive modeling of epidemiological dynamics under changing conditions.

The data for the experiments consisted of the following daily time series variables collected in Ontario, Canada, all of which are publicly available and de-identified from open sources (citations given for each variable below). This is a similar dataset to another study [21]. Here is a more detailed description of the dataset:

1. Number of confirmed COVID-19 cases: The number of confirmed COVID-19 cases was collected daily from various public health units across Ontario [22].
2. Effective reproduction number: An epidemiological property that represents the average number of infections that one case generates from a disease over a certain time period [23] in the population, estimated by Ontario Health [24].
3. Cumulative number of fully vaccinated individuals: Ontario Health collected the cumulative number of fully vaccinated individuals. To be *fully vaccinated*, an individual requires two doses of an Ontario-approved COVID-19 vaccine.
4. Google mobility measurements: Google collected mobility data as part of their Community Mobility Reports [25]. This data shows where individuals spend time compared to a baseline day. The baseline day is the median value for the five weeks from January 3, 2020, to February 6, 2020. For example, the *workplace* category represents individuals visiting their place of work. Additionally there are retail and recreation, grocery and pharmacy, parks, transit stations, and residential mobility measurements in this dataset.

The variables entering the causal discovery step of CaReBayes were confirmed COVID-19 cases, $R_{t}$, cumulative fully vaccinated individuals, and the Google mobility variables: retail and recreation, grocery and pharmacy, parks, transit stations, workplaces, and residential mobility. The forecasting target was confirmed COVID-19 cases.

The real-world dataset experiment was conducted to test whether CaReBayes can detect regime changes and forecast more accurately than baseline methods. The model selection procedure of the CaReBayes framework determined that the most appropriate number of regimes for the COVID-19 dataset was two ($N_{K}$) with one transition ($N_{C}$). The estimated regime switch occurs on July 10th.

The regime classification phase for the forecasting test data predicted that the selected regime from July 10 to September 28 (end of the training data) remained the same throughout the test data. See Table 1 for summary statistics on the time series data.

**Table 1:** Summary statistics of COVID-19 data, with the mean (μ), standard deviation (σ), minimum and maximum values reported.

Variable	μ	σ	Minimum	Maximum
COVID-19 cases	396.08	340.46	32.00	1599.00
Effective reproduction number	1.10	0.30	0.73	2.98
Fully Vaccinated Individuals	$1.66\times 10^{6}$	$3.35\times 10^{6}$	0.00	$1.05\times 10^{7}$
Retail and Recreation (mobility)	−29.33	15.33	−82.00	−3.00
Grocery and Pharmacy (mobility)	−6.75	12.68	−68.00	41.00
Parks (mobility)	71.55	71.25	−47.00	276.00
Transit Stations (mobility)	−50.53	11.44	−81.00	−16.00
Workplaces (mobility)	−38.44	19.11	−86.00	0.00
Residential (mobility)	13.41	7.03	−1.00	36.00

## Results

Our experiments with simulated and real-world data were conducted to address the following: first, whether an automated procedure accurately estimates regime-dependent causal structure; next, whether a method reliably classify future observations into the correct regime; and finally, whether there is empirical evidence that CaReBayes achieves superior forecasting performance compared to suitable benchmarks.

### Simulation experiments

As mentioned previously, we required simulated time series to assess the ability of CaReBayes for (1) regime-dependent causal discovery and (2) regime classification of future data.

#### Regime-dependent causal discovery.

Across all three simulation systems, the grid-search selection procedure correctly identified both the true number of regimes ($N_{K}$) and the number of regime transitions ($N_{C}$). Table 2 reports the mean causal recovery performance across Monte Carlo simulations. For all systems, CaReBayes achieved 100% accuracy in recovering both $N_{K}$ and $N_{C}$, demonstrating that the model selection procedure reliably identifies the underlying regime structure.

**Table 2:** Structural recovery and edge stability across Monte Carlo simulations. Regime number accuracy ($N_{K}$) and regime switch detection ($N_{C}$) are reported as the proportion of simulations in which the true values were correctly identified. Edge stability is reported using the mean (μ) and standard deviation (σ) of causal discovery accuracy across Monte Carlo simulations.

System	$N_{K}$ Accuracy (%)	$N_{C}$ Accuracy (%)	Edge Stability (%)	
			μ	σ
1	100	100	95.08	2.65
2	100	100	99.00	2.50
3	100	100	99.33	2.28

Accurate recovery of $N_{K}$ and $N_{C}$ is critical, as the regime-aware Bayesian structural autoregressive models are conditioned on this segmentation. These results therefore indicate that the proposed framework can consistently recover the correct regime configuration across systems with varying dynamics.

To assess the robustness of the inferred causal structures, we further evaluated edge stability across Monte Carlo simulations. Edge stability is defined as the proportion of correctly recovered edges relative to the ground truth across repeated runs. In System 1, which contains 14 total edges across both regimes, there was an edge stability of 95.08%. In Systems 2 and 3, the edge stability was about 99%.

These results indicate that the inferred causal graphs are robust to stochastic variation in the data and that the proposed framework consistently recovers the underlying causal structure across repeated realizations.

### Test data regime classification

To infer the regime for each test sample, we applied the regime classification procedure. This approach selects the regime that produces the lowest cumulative forecasting error on the observed data over a window.

More specifically, for each test sample, we computed the total squared error between the observed values and the predictions generated by each regime-specific model. The sample was then assigned to the regime with the minimum error over a sliding window, classifying each time step into a regime.

The rolling window size was selected empirically (with the training set) to produce stable, contiguous regime segments. Several candidate values of *w* were evaluated in increasing order, and the selected value was chosen to balance high regime classification accuracy with stable and contiguous regime assignments. In our simulations, systems with lower one-step (window size of 1) classification accuracy generally required larger window sizes. For example, as shown in Table 3, System 1 has a mean classification accuracy of 90.16% with a window size of 1 required a window of size 5, whereas System 2, with a lower accuracy of 64.66% at window size 1, required a larger window of 35 to achieve stable classification. We did not fine-tune the window size, and therefore more optimal choices may exist.

**Table 3:** Regime classification accuracy, with the mean (μ) and standard deviation (σ) across fifty Monte Carlo simulations for each system.

System	Window Size	Classification Accuracy	
		μ (%)	σ (%)
1	1	90.16	2.11
	5	98.56	0.51
2	1	64.66	3.24
	35	91.40	4.44
3	1	79.53	2.41
	10	97.58	1.44

Nevertheless, we correctly classified a mean of 95.85% of the test samples across systems (see Table 3), demonstrating the effectiveness of the proposed approach in distinguishing between regimes based on causal structure-informed classification. As mentioned previously, this step is crucial, as the forecasts produced by the regime-aware Bayesian Structural Autoregressive models depend on accurate regime estimation.

For applications where the true regime labels are unknown, the rolling window size *w* cannot be selected using regime classification accuracy directly. Instead, *w* can be treated as a hyperparameter and selected using a validation-based criterion. In this setting, regime-dependent causal discovery can be performed on the training data to estimate candidate regime structures, and a grid of candidate window sizes can then be evaluated on this training period. Then the selected value of *w* can be chosen based on forecasting error, the stability of the inferred regime assignments, or a combination of both. While this procedure does not provide a fully independent validation criterion, it offers a practical approach for selecting *w* when ground-truth regime labels are unavailable. Since the selected regime determines which regime-specific Bayesian model is used for forecasting, different choices of *w* may affect both regime classification accuracy and downstream forecasting performance.

#### Uncertainty quantification.

The CaReBayes framework produces a posterior predictive distribution for each forecast. This posterior predictive distribution provides a measure of forecast uncertainty. In the experiments, uncertainty was evaluated using 95% posterior predictive intervals.

Let $y_{t}$ denote the observed value of the target variable at time *t* ($X_{t}^{2}$ in simulated data studies), and le*t* $L_{t}$ and $U_{t}$ denote the lower and upper bounds of the 95% prediction interval, respectively. The empirical prediction interval coverage is defined as


$$\begin{array}{c} \frac{1}{T}\sum_{t=1}^{T}𝕀\left(L_{t}\leq y_{t}\leq U_{t}\right), \end{array}$$ (13)

where 𝕀(·) is an indicator function equal to one if the observation lies within the prediction interval and zero otherwise. For a nominal 95% prediction interval, a well-calibrated model should have coverage close to 0.95. The mean predictive interval width is defined as


$$\begin{array}{c} \frac{1}{T}\sum_{t=1}^{T}\left(U_{t}-L_{t}\right). \end{array}$$ (14)

While wider intervals usually increase coverage, they also indicate greater forecast uncertainty. Therefore, interval quality depends on achieving high coverage without producing unnecessarily wide intervals.

To compare interval width relative to the variability of the target variable, the normalized prediction interval width is computed by dividing the mean predictive interval by the standard deviation of the observed target variable, $\sigma_{y}$, over the test period. This metric expresses the mean interval width in units of the empirical standard deviation of the target variable, $X_{t}^{2}$.

The Winkler score provides a combined measure of interval width and coverage. For a (1−α) prediction interval, the Winkler score at time *t* is defined as


$$\begin{array}{c} W_{t}=\left\{ \begin{array}{cc} U_{t}-L_{t}, & \text{if }L_{t}\leq y_{t}\leq U_{t},\\ (U_{t}-L_{t})+\frac{2}{\alpha }(L_{t}-y_{t}), & \text{if }y_{t}<L_{t},\\ (U_{t}-L_{t})+\frac{2}{\alpha }(y_{t}-U_{t}), & \text{if }y_{t}>U_{t}. \end{array}\right. \end{array}$$ (15)

The mean Winkler score is then


$$\begin{array}{c} \frac{1}{T}\sum_{t=1}^{T}W_{t}. \end{array}$$ (16)

A lower Winkler scores indicates better uncertainty estimates, since the score rewards narrower prediction intervals but penalizes observations that are outside the interval.

Table 4 shows that CaReBayes produces well-calibrated uncertainty estimates across all three systems. The average 95% prediction interval coverage is 0.950, 0.949, and 0.948 for Systems 1, 2, and 3, respectively, which closely matches the nominal 95% coverage level. This indicates that the posterior predictive intervals capture the observed target values at approximately the expected rate across the Monte Carlo simulations.

**Table 4:** Uncertainty quantification across Monte Carlo simulations using 95% posterior predictive intervals (PI) over the test set. Note that μ and σ denote the mean and standard deviation across fifty Monte Carlo simulations.

System	Metric	μ	σ	Minimum	Maximum
1	95% PI Coverage	0.950	0.020	0.855	0.985
	95% PI Width	4.077	0.175	3.658	4.484
	95% PI Winkler Score	4.946	0.500	4.260	7.587
	Normalized PI Width	2.338	0.343	1.490	2.872
2	95% PI Coverage	0.949	0.014	0.915	0.980
	95% PI Width	3.967	0.112	3.719	4.156
	95% PI Winkler Score	4.737	0.322	4.120	5.483
	Normalized PI Width	3.401	0.157	3.093	3.786
3	95% PI Coverage	0.948	0.019	0.890	0.975
	95% PI Width	3.969	0.120	3.757	4.264
	95% PI Winkler Score	4.890	0.562	3.962	7.420
	Normalized PI Width	2.860	0.195	2.572	3.445

The mean prediction interval widths are also similar across the three systems, ranging from 3.967 to 4.077. However, the normalized interval widths differ across systems, indicating that the same interval width can represent different levels of uncertainty relative to the variability of the target variable. System 1 has the smallest normalized width, with an average value of 2.338, while System 2 has the largest normalized width, with an average value of 3.401. The Winkler scores are lowest for System 2 and highest for System 1, although the values are broadly similar across systems. Overall, these results suggest that CaReBayes achieves prediction intervals that are close to the desired 95% coverage level while maintaining relatively stable interval widths across the fifty Monte Carlo simulations.

### Forecasting performance

Finally, we evaluate the forecasting performance of CaReBayes by comparing it to both a baseline vector autoregressive (VAR) model and Markov-switching VAR (MS-VAR) model. Both benchmark models were implemented with the *statsmodels* library [26]. The MS-VAR model does not have an automated method to determine the optimal number of regimes in the dataset, so this was provided by the CaReBayes regime-based causal discovery phase.

#### Simulated data.

Forecasting performance was evaluated across Monte Carlo experiments with multiple independent replications for each synthetic system to assess robustness under different realizations. For each simulation, CaReBayes was fit on the training data, the regime at time *t* was classified, and one-step-ahead forecasts of the target variable *X*^2^ at time *t* + 1 were produced (as were the benchmark models).

Performance was evaluated using three common forecasting metrics: coefficient of determination (*R*^2^), mean absolute error (MAE), and root mean squared error (RMSE). Table 5 reports the mean (μ) and standard deviation (σ) of each metric across Monte Carlo runs. It is clear that CaReBayes consistently outperforms both the baseline VAR and MS-VAR models across all simulated systems.

**Table 5:** Forecasting performance for fifty Monte Carlo simulated systems, with the mean (μ) and standard deviation (σ) of each system.

System	Model	R^2^		MAE		RMSE	
		μ	σ	μ	σ	μ	σ
1	CaReBayes	0.846	0.056	0.823	0.047	1.034	0.060
	Markov-Switching VAR	0.834	0.064	0.848	0.059	1.071	0.074
	VAR	0.720	0.103	1.105	0.062	1.399	0.079
2	CaReBayes	0.263	0.075	0.806	0.042	1.009	0.047
	Markov-Switching VAR	0.218	0.066	0.834	0.038	1.040	0.044
	VAR	0.218	0.064	0.834	0.037	1.039	0.042
3	CaReBayes	0.518	0.079	0.815	0.051	1.026	0.067
	Markov-Switching VAR	0.469	0.103	0.858	0.096	1.077	0.111
	VAR	0.315	0.065	0.982	0.057	1.227	0.066

Notably, CaReBayes achieves higher *R*^2^ and lower MAE and RMSE values, demonstrating its ability to exploit regime-aware causal structure for improved forecasting performance.

#### Real-world COVID-19 experiments.

We further evaluate CaReBayes using real-world data from COVID-19 data collected in Ontario, Canada. The complete dataset spans from March 13, 2020 to November 17, 2020 and consists of the number of new COVID-19 cases, the effective reproduction number, the total number of fully vaccinated individuals, and mobility factors that measure the movement of individuals across Ontario.

The forecasting target in this experiment is the number of *confirmed COVID-19 cases*. We perform one-step-ahead forecasting on the final 50 days of the time series, simulating a realistic infectious-disease forecasting scenario under potential regime shifts. Table 6 reports forecasting performance based on mean absolute error (MAE), root mean squared error (RMSE), and the coefficient of determination (*R*^2^).

**Table 6:** Forecasting performance comparison for real-world COVID-19 time series data.

Model	R^2^	MAE	RMSE
CaReBayes	0.836	98.60	117.64
Markov-Switching VAR	0.707	125.91	160.02
VAR	0.752	115.86	144.58

We set the maximum lag to $\ell_{\max}=1$ for consistency with the benchmark VAR and MS-VAR models and to provide a controlled illustration of the CaReBayes framework. Consequently, the COVID-19 results should be interpreted as identifying one-day lagged conditional relationships under the RPCMCI assumptions, rather than as a complete epidemiological causal model. Longer delay structures, such as 7-day, 14-day, or variable-specific lags for vaccination and mobility, may reveal additional relationships and should be considered in future empirical applications.

**Fig 4:**
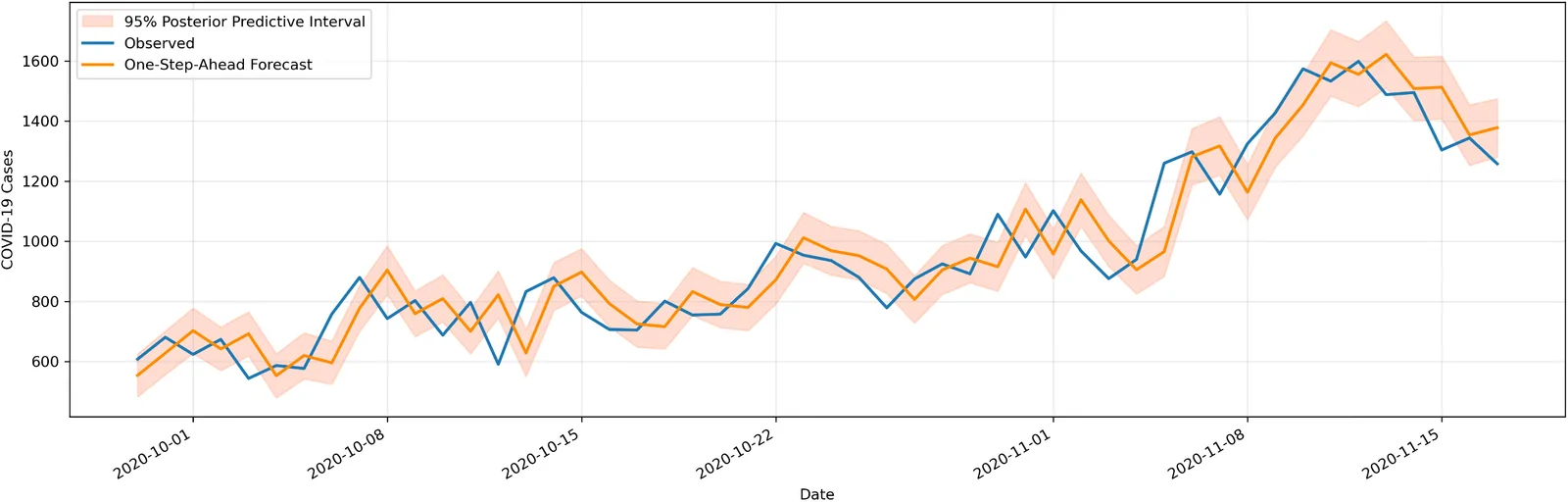
The CaReBayes forecast with 95% posterior predictive interval for the COVID-19 data. The observed, one-step-ahead forecast and posterior predictive interval values over the 50-day evaluation period.

Fig 4 presents the one-step-ahead forecasts and posterior predictive intervals produced by CaReBayes over the 50-day evaluation period compared to the observed values. The forecasts are dominated by persistence in the outcome, reflecting the strong dependence of the current number of COVID-19 cases on the previous day’s value. The additional estimated causal parents, transit-station and workplace mobility, provide conditional adjustments to this autoregressive component and may be more informative for intervention analysis than for short-horizon prediction.

CaReBayes achieves the best performance across all metrics, highlighting its ability to adapt to structural changes in the data-generating process. The Markov-Switching VAR (MS-VAR) model performed worse than VAR, showing that it was not able to accurately learn the regime-dependent structure of this problem.

## Discussion

The experimental results of this study reveal the importance of modeling regime-dependent causal structure for some time series forecasting tasks. By explicitly capturing the evolving causal relationships in non-stationary time series, our approach provides causal insights into the dynamics governing complex systems (causal interpretability). The forecasting results show that our approach is also valuable for accurate forecasting in these systems.

After assigning each test example to a regime, we utilize the corresponding regime-specific causal Bayesian model to generate forecasts for future time steps. These forecasts incorporate the regime-dependent causal structure learned during training, reflecting the underlying data-generating process of each regime.

**Fig 5:**
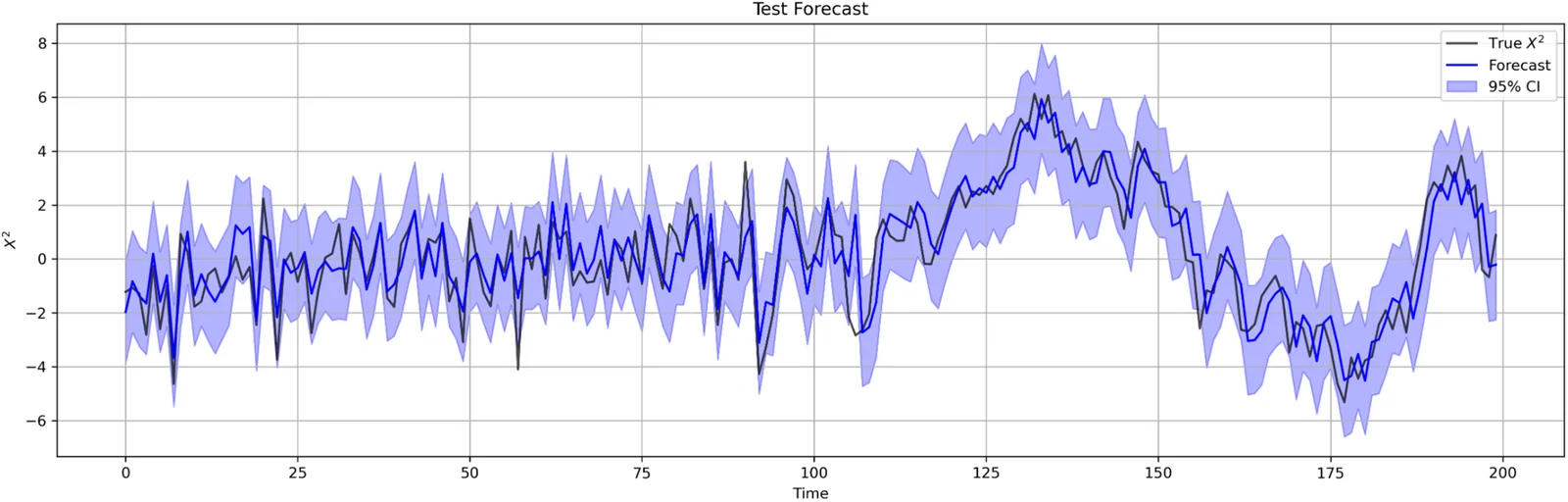
The CaReBayes forecast with 95% posterior predictive interval for the simulated data. The forecast for system 1, Monte Carlo simulation 3 test data. The true regime switch occurs at time step 105 between regime 0 and 1.

Additionally CaReBayes provides an estimate of the uncertainty in the forecast (see Fig 5 and Table 4). The uncertainty estimates produced by the Bayesian approach enhance reliability and trust for downstream tasks, such as decision-making under uncertainty and anomaly detection.

### Explainability

The explainability of a prediction has recently become an important part of ML research [27]. We want to know *why* the ML model made a certain prediction [28]. In [29], explainability is defined as “the degree to which a human can understand the cause of a decision or the degree to which a human can consistently predict the model’s result”, this the opposite of a *black-box* model [30].

In order to have explainability from our forecasting model, we want more than just a predicted future outcome, we want to know what factors are driving the spread so that we can potentially intervene to slow the spread. This is what is meant by having actionable insights. If we want variables that we can intervene on we are asking for the causal factors of the disease spread.

Beyond standard time series forecasting, CaReBayes provides causal insights, identifying the underlying mechanisms driving time series evolution in each regime. This capability distinguishes CaReBayes from traditional time series models, which typically lack interpretability and cannot identify the directed causal links between variables.

**Fig 6:**
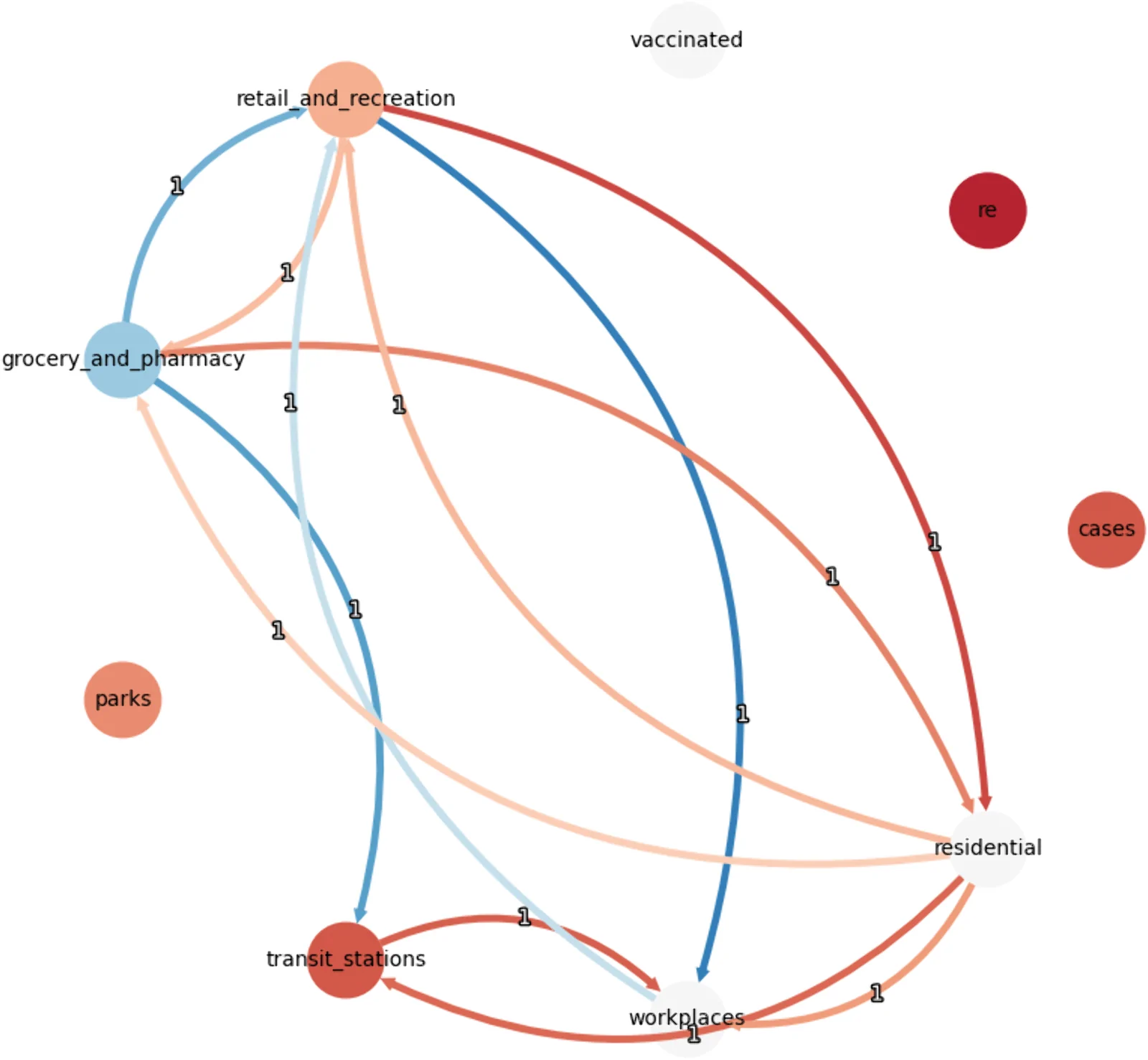
COVID-19 data, regime 1 causal graph. The estimated causal graph from the regime-dependent causal discovery phase. Note the target *cases* has no causal arrows, indicating that no strong causal drivers were detected under the modeling assumptions (beyond lagged values of itself).

**Fig 7:**
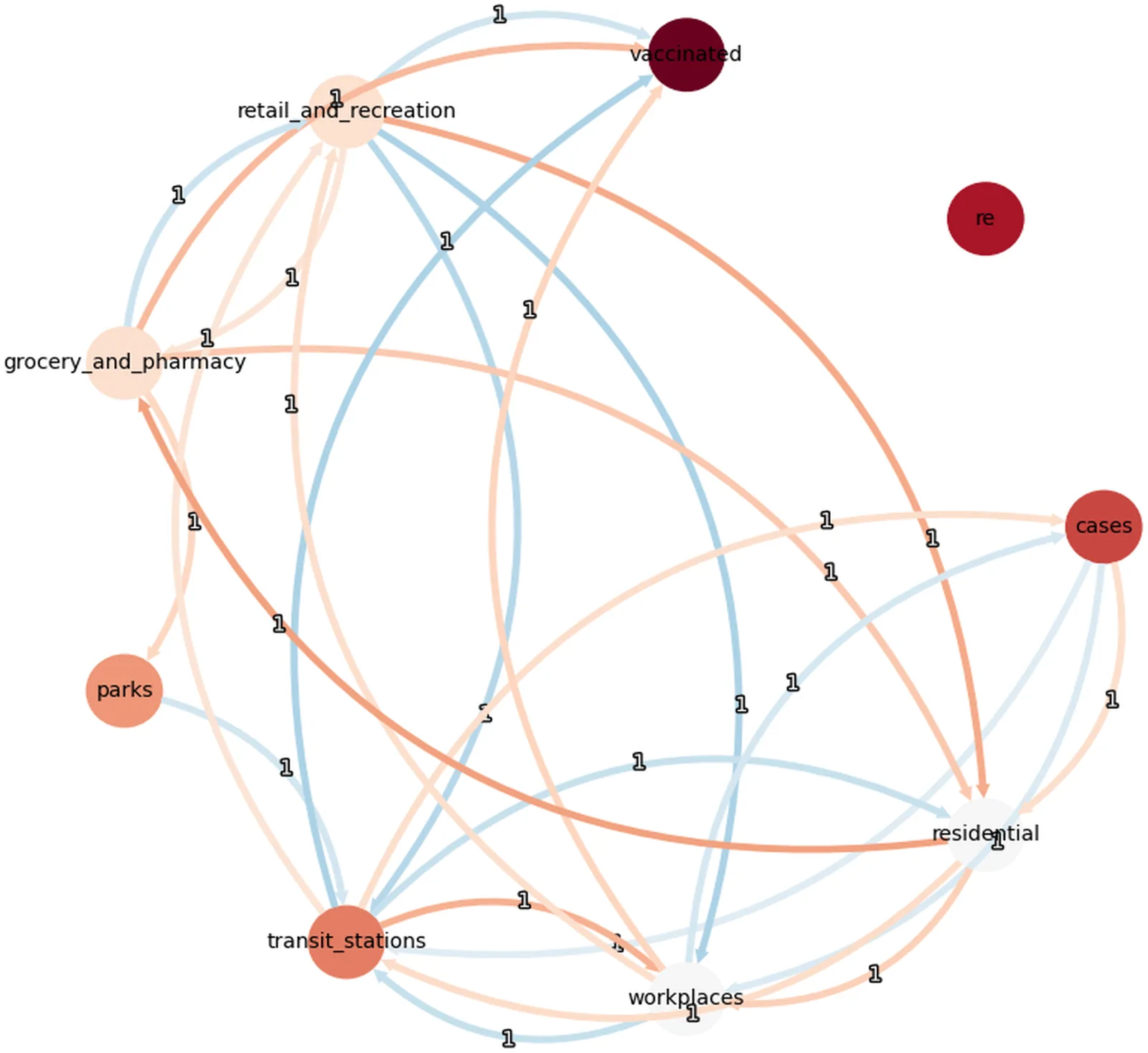
COVID-19 data, regime 2 causal graph. The estimated causal graph from the regime-dependent causal discovery phase. Notice the target *cases* has two causal drivers: the movement of residents in transit stations and workplaces.

Regarding our experiment on real-world COVID-19 data, Figs 6 and 7 show the two summary causal graphs estimated from the COVID-19 data. These graphs show the regime-dependent causal structure between variables with directed arrows. The relationship can be either positive (red) or negative (blue). Each edge also has *lag 1* associated with it. This is because for the regime causal discovery in training data, the maximum lags to consider was set to one day. This was done to simplify the experiments with CaReBayes, VAR, and Markov-Switching VAR, although it could be set to any number of past lags.

Note that in the empirical COVID-19 experiment, a directed edge $X_{t-\ell }^{i}\to X_{t}^{j}$ should be interpreted as a regime-specific lagged parent relationship selected by RPCMCI under the assumed lag structure and conditional independence test, rather than as a definitive interventional causal effect.

Focusing on the forecasting target, confirmed cases, Regime 1 contains no selected lagged parents of cases other than its own past values under the RPCMCI specification. This should not be interpreted as evidence that other epidemiological variables have no causal effect on cases. Rather, it indicates that no additional one-day lagged parent was selected by the conditional independence procedure in this regime.

In Regime 2 (starting on July 10th), lagged transit-station mobility and workplace mobility are selected as parents of confirmed cases. These links indicate regime-specific lagged conditional dependencies that are consistent with a structural causal interpretation under the assumptions of RPCMCI. However, because the COVID-19 analysis is observational, these estimated links should be interpreted as putative causal relationships rather than definitive interventional effects.

The (hypothetical) causal structures revealed in this COVID-19 data help us understand *why* the spread of the virus and other epidemiological factors change over time. The CaReBayes framework is a step toward forming a *theory* of the system under study. This theory can be refined over time with knowledge from domain experts and the collection of more data. In this analysis, determining that during Regime 1 (March 13th, 2020 to July 9th, 2020) the spread of COVID-19 was causally driven only by its past values provides valuable information to healthcare professionals about how the disease spreads and how to slow it.

As we saw, in Regime 2 the causal drivers shift to include the movement of residents in transit stations and workplaces. The signs of the estimated coefficients suggest that, within this regime-specific model, lagged transit-station mobility is positively associated with future confirmed cases, while lagged workplace mobility is negatively associated with future confirmed cases. These signs should not be interpreted as the effect of intervening on mobility without additional assumptions or validation.

### Limitations

Regarding the COVID-19 data, it is important to note that these results do not establish that the regime-dependent causal structures identified by CaReBayes are correct. The causal structure algorithm used (PCMCI) used a linear detection method, while the true dynamics of the time series may be nonlinear (though PCMCI can be configured to use nonlinear detection methods). Moreover, constraint-based causal discovery algorithms, such as PCMCI, assume causal faithfulness and sufficiency [31].

In observational settings such as epidemiological time series, causal identifiability is inherently limited. The validity of inferred causal relationships depends on assumptions such as causal sufficiency (no unobserved confounders), faithfulness, and correct model specification. Violations of these assumptions may lead to spurious or missed causal links. If the causal discovery step of CaReBayes selects only lagged values of the target variable as parents, then the resulting regime-specific model is effectively autoregressive for that target. This distinction is especially important in the COVID-19 application, where Regime 1 is autoregressive under the one-day lag specification, whereas Regime 2 includes mobility-related lagged parents of cases.

Moreover, while the estimated causal graphs encode conditional dependencies that are consistent with a causal interpretation, they do not, on their own, establish interventional effects. Estimating the effect of an intervention would require additional assumptions or experimental data. Therefore, the causal structures identified by CaReBayes should be viewed as informative and interpretable summaries of the data-generating process that can guide further investigation, rather than as fully identified causal mechanisms.

Nevertheless, this causal analysis provides critical insight into what might be driving the spread of COVID-19. Domain experts can use this information to help inform policy decisions. In contrast, the benchmark forecasting models (VAR and Markov-switching VAR) offer no such causal insights into the system under study.

Another limitation is that the inferred regime structure may be sensitive to the specified search range for $N_{K}$ and $N_{C}$. CaReBayes selects the best $\left(N_{K},N_{C}\right)$ configuration from a finite candidate grid using AICc, so the selected regimes, causal graphs, and forecasting performance are conditional on the range of configurations considered. A narrow search range may exclude the true regime structure, while a broad range will increase computational cost. Although AICc penalizes complexity, sensitivity analysis over plausible search ranges should be conducted when computationally feasible, and future work should consider model averaging or Bayesian selection over regime configurations.

In this study, a fixed lag (e.g., ℓ=1) is assumed, simplifying the process of causal discovery. Considering larger lag windows increases the complexity of the model, making causal discovery more challenging and potentially reducing estimation reliability. Selecting an incorrect lag structure (lag misspecification) will impact both the inferred causal structure and forecasting performance.

The framework will also be less effective when regime changes occur very frequently or when regimes persist only for short periods of time. The regime classification step relies on cumulative prediction errors over a rolling window, so short regimes may not provide enough observations for reliable classification. Smaller window sizes can respond more quickly to regime switches but may produce noisy regime assignments, while larger windows provide more stable classifications but may delay detection of rapid changes. As a result, CaReBayes is best suited to settings where regimes persist long enough for regime-specific causal structures to be estimated and recognized.

## Future work

In its current form, the framework assumes that the regime at the next time step will be the same as the most recent one and therefore uses the corresponding Bayesian Structural Autoregressive Model for forecasting. As a result, even with perfect regime classification, the technique will misclassify at least one time step during regime changes.

Future extensions should also address the challenge of detecting the emergence of entirely new regimes not observed during training, enabling adaptive causal discovery and real-time model updating.

There is also work to be done with the various hyperparameters that are part of this framework. The grid search approach to finding the ground truth number of regimes and transitions worked well in this study, but it is a computationally intense method. Other methods such as genetic algorithms could search for optimal hyperparameters.

As mentioned, in our simulated study, we implemented CaReBayes with a linear version of PCMCI, since the simulated data was linear. Studying this regime-dependent causal structure with nonlinear data is a critical next step.

## Conclusion

In this study, we introduced a regime-aware causal Bayesian forecasting framework, *CaReBayes*. We demonstrated that this approach can automatically estimate regime-dependent causal structures, guided by a novel grid search over both the number of regimes and transitions. These estimated causal structures were then used as scaffolds for Bayesian autoregressive models to classify future data into the current regime by applying a sliding window over the prediction errors.

We compared our framework against two benchmark forecasting models: a baseline Vector Autoregression (VAR) model, which ignores regime structure, and a Markov-Switching Vector Autoregression (MS-VAR), a regime-dependent time series forecasting, on synthetic and real-world time series. Our experimental results show that *CaReBayes* outperformed both forecasting models across all evaluation metrics.

Beyond forecasting performance, our framework also provides interpretable causal insights, revealing the underlying mechanisms that drive regime-dependent dynamics. These insights can be leveraged to guide interventional analysis and support more informed decision-making. In addition, the Bayesian formulation enables uncertainty quantification in both model parameters and forecasts.

Overall, *CaReBayes* provides a unified framework for understanding and forecasting non-stationary time series, bridging the gap between causal discovery and forecasting. This makes it a useful tool for analyzing complex systems in which both accurate forecasts and interpretable structure are essential.
